# Nurses' Knowledge, Perceived Practice, and their Associated Factors regarding Deep Venous Thrombosis (DVT) Prevention in Amhara Region Comprehensive Specialized Hospitals, Northwest Ethiopia, 2021: A Cross-Sectional Study

**DOI:** 10.1155/2022/7386597

**Published:** 2022-03-16

**Authors:** Senay Yohannes, Tarkie Abebe, Kidist Endalkachew, Destaw Endeshaw

**Affiliations:** ^1^Department of Surgical Nursing, School of Nursing, College of Medicine and Health Science, University of Gondar, Gondar, Ethiopia; ^2^Department of Comprehensive Nursing, School of Nursing, College of Medicine and Health Science, University of Gondar, Gondar, Ethiopia; ^3^Department of Surgical Nursing, School of Nursing and Midwifery, College of Medicine and Health Science, Bahir Dar University, Bahirdar, Ethiopia

## Abstract

**Introduction:**

Deep venous thrombosis is a preventable and treatable cause of death among hospitalized patients. Nurses' knowledge and proper assessment can play a major role in improving deep venous thrombosis prevention care.

**Objective:**

To assess the knowledge, practice, and associated factors towards deep venous thrombosis prevention among nurses working at Amhara region hospitals.

**Methods:**

Institutional-based cross-sectional study was conducted among nurses working at Amhara region comprehensive specialized hospitals, Northwest, Ethiopia, from April 1 to 30, 2021. A simple random sampling technique was used to select 423 samples. A structured pretested self-administered questionnaire was used to collect data. Data were entered in epi-info version 7, analyzed using SPSS version 25, and presented by frequencies, percentages, and tables. Bivariable and multivariable logistic regression was computed, and *P* value < 0.05 was considered to identify statistically significant factors.

**Result:**

Good knowledge and practice of nurses towards DVT prevention were 55.6% and 48.8%, respectively. Working at the medical ward [AOR 3.175, 95% CI (1.42, 7.11)], having a BSc degree [AOR = 3.248(1.245, 8.469)], Master's degree [AOR = 3.48, 95% CI (1.22, 9.89)], obtaining a formal training about deep venous thrombosis [AOR = 1.59; 95% CI (1.03, 2.47)], and working experience of ≥11 years [AOR = 2.11; 95% CI (1.07, 4.16)] were associated with good knowledge of nurses on the prevention of deep venous thrombosis. While having good knowledge about deep venous prevention AOR = 1.75; 95% CI (1.15, 2.65)] and working experience ≥11 years [AOR = 3.44; 95% CI (1.45, 8.13)] were significantly associated with nurses' practice about deep venous thrombosis prevention.

**Conclusion:**

Knowledge and practice of the nurses regarding the prevention of deep venous thrombosis were found to be inadequate. Therefore, providing training, creating a conducive environment for sharing of experience, and upgrading the academic status of nurses are measures to scale up the knowledge and practice of nurses regarding deep venous thrombosis prevention.

## 1. Background

Deep venous thrombosis (DVT) is a common and serious pathology among hospitalized patients, which is a potentially preventable and treatable health problem that contributes to patients' morbidity and mortality [1].

Both deep vein thrombosis (DVT) and pulmonary embolism (PE) are the elements of venous thromboembolism (VTE), become a major public health concern of population of the USA affecting more than 900,000 people, and nearly 60,000–100,000 of them are died because of venous thromboembolism (VTE) each year [2].

The European Union also experiences a substantial venous thromboembolism burden of nearly 684,019 deep venous thrombosis, 434,723 pulmonary embolisms, and 610,138 post-thrombotic syndrome events that occur annually and cost billions of dollars each year [3].

According to the center for disease control and prevention (CDC), venous thromboembolism is the 5^th^ most reason for unplanned hospital readmissions of patients after surgery, and from this, 70% of cases of hospital-acquired venous thromboembolism (HA-VTE) are preventable through preventive measures [2].

Based on the systematic review done in Africa, the prevalence of pulmonary embolism (PE) in medical patients ranges up to 61.5%, with a mortality rate between 40% and 69.5%. And the case-fatality rate of pulmonary embolism (PE) after surgery was 60% [4].

A study conducted on assessing nurses' knowledge and practice about venous thromboembolism prevention for cancer surgery patients in Aswan Oncology Center of Egypt revealed that a total score of nurses' knowledge and observed practice level regarding prevention was unsatisfactory [5].

A study conducted in teaching hospitals of Ethiopia shows that out of 200 medically admitted patients, 186 (93%) of them have at least two risk factors for VTE development. Only 75 (40%) patients received thromboprophylaxis, and VTE has prevented in 61 (32.8%) patients who received prophylaxis [6].

Venous thrombus embolism accounts for almost 10% of all hospital deaths, and over half of VTE incidents are hospital-acquired. Appropriate preventive practice (e.g., pharmacological and mechanical prophylaxis) can significantly reduce the incidence of VTE by 70% for both medical and surgical patients [7].

Deep venous thrombosis is considered the third most common cardiovascular condition following myocardial infarction and stroke, and it is a growing public health problem with 26.4% of recurrent after the patients have been diagnosed, and this results in further cost of treatment for patients and intensifies hospital's burden [8].

Deep venous thrombosis (DVT) prevention includes the three arms, pharmacological, mechanical, and general care (early mobilization, exercising, and hydration) of hospitalized patients can reduce the incidence of DVT in both medical and surgical patients significantly [9].

Nurses are key components to assess and recognize risk factors of deep venous thrombosis of patients in the hospital care setting. When sufficient knowledge along with proper patient care including graduated compression stockings, administration of the correct dose of an anticoagulation agent with careful assessment, and monitoring of risk factors by nurses help to minimize the burden of DVT and its complication [10, 11].

Studies have shown that having a poor level of knowledge and expressed practice of nurses on prevention of deep venous thromboembolism could increase hospitalization and ultimately leads to poor health care outcome [9, 12].

Despite the advance in medical care, the presence of effective strategies, and standard guidelines, deep vein thrombosis (DVT) prevention is not possible as it is needed and expected. So, this study was aimed to assess the actual gap in knowledge, practice, and its associated factors of DVT prevention among nurses working at Amhara region comprehensive specialized hospitals, Northwest, Ethiopia.

## 2. Method and Materials

### 2.1. Study Design, Area, and Period

An institution-based cross-sectional study was conducted from April1 30, 2021, at five comprehensive specialized hospitals of the Amhara region, Northwest, Ethiopia.

The study was conducted in Amhara Regional State Referral Hospitals, Northwest, Ethiopia. There are five government comprehensive specialized hospitals found in Amhara regional state, Northwest, Ethiopia such as University of Gondar Comprehensive Specialized Hospital (UoGCSH), Felegehiwot Comprehensive Specialized Hospital (FHCSH), Tibebegion Specialized Teaching Hospital (TGSTH), Debre Markos Comprehensive Specialized Hospital (DMCSH), and Debre Tabor Comprehensive Specialized Hospital (DTCSH). All hospitals provide outpatient and inpatient services for more than 22,000,000 people living in their catchment area. Currently, these hospitals have 1682 nurses, and the total number of nurses who are working in surgical, medical, ICU, emergency, and Gyn-obs wards were around 728.

### 2.2. Source Population and Study Population

Source populations were all nurses who were working in medical, surgical, emergency, ICU, and Gyn-obs wards of Amhara Region comprehensive specialized hospitals, Northwest, Ethiopia. Whereas all nurses who were working in selected units or wards (medical, surgical, emergency, ICU, and Gyn-obs) at UoGCSRH, FHCSH, TGSTH, DTCSH, DMCSH, and available during the data collection period are included in the study.

### 2.3. Inclusion and Exclusion Criteria

All nurses working in surgical, medical, ICU, emergency, and Gyn-obs units of Amhara region Comprehensive Specialized hospitals, Northwest, Ethiopia, during the study period were included in the study, while those who are working as matron and an administrator were excluded in the study.

### 2.4. Sample Size Determination and Sampling Technique

#### 2.4.1. Sample Size Determination

The sample size was calculated using the single population proportion formula. Since there was no similar published study found in our country addressing knowledge and practice on prevention of DVT, so considered the proportion of knowledge and practice (*p*) as 50% using the following formula

(1) $$\begin{array}{c} n=\frac{{\left(Z\alpha /2\right)}^{2}p\left(1-p\right)}{d^{2}}, \end{array}$$

where *n* = minimum sample size required for the study, *Z* = standard normal distribution (*Z* = 1.96) with CI of 95% and *α* = 0.05, *P* = population proportion (*p*=0.5), *d* = is a tolerable margin of error (*d* = 0.05), and *n* = 1.96(1.96) (0.5(1–0.5))/0.05(0.05) = 384. By adding a 10%, nonresponse rate the final sample size was 423.

#### 2.4.2. Sampling Technique

A stratified simple random sampling technique was employed to recruit the required participants for the study. First, we stratified participants from each hospital and working ward/unit, and then we allocated the required sample for each stratum proportionally. Finally, we selected study participants from each stratum by simple random sampling.

### 2.5. Operational Definitions

Good knowledge: respondents were labeled to have “good knowledge of DVT prevention” if they score the mean score or above, on the closed-ended knowledge questions of DVT prevention.

Good practice: respondents were labeled to have “good practice of DVT prevention” if they score the mean score or above, on the closed-ended knowledge questions of DVT prevention.

### 2.6. Data Collection Tool and Procedure

Data were collected using a self-administered structured questionnaire to obtain information from participants. The questionnaire has three parts, the first section is regarding the sociodemographic characteristics of nurses and included 10 questions. The second section consists of 34 questions regarding knowledge of nurses on DVT prevention with 3 choices (true, false, and I do not know). The last section consisted of 13 questions concerning the practices of nurses on DVT prevention with 3 points Likert scale (always = 2, sometimes = 1, and never = 0), Which were adopted from a study conducted in the Near East University hospital, North Cyprus, Turkey [9].

Eligible study participants were approached in each ward unit. Participants were provided with appropriate information about the study, then informed consent was be obtained to assure their willingness to participate in the study. Five trained BSc nurses collected the data, and five trained MSc nurses closely followed the data collection process.

The instruments were distributed among the study population, after guarantying their willingness to take part in the study, and then it was collected by the data collectors after completion. During data collection, data collectors and supervisors followed the recommended precautions to prevent COVID-19.

### 2.7. Data Quality Assurance

A self-administered structured questionnaire was prepared, and training was given for both collectors and supervisors about the concept of the questionnaire and the rights of the participants two days before the actual day of data collection. Moreover, the tool was pretested in 10% of the total sample size at Dessie comprehensive specialized hospital a week before the actual data collection period was conducted. Based on the finding, necessary modifications were done to the wording and phrases. The reliability of the tool used for measuring the dependent variable was 0.786 and 0.760 for knowledge and practice, respectively. There was regular supervision, spot-checking, and reviewing the completed questionnaire to maintain data quality. Data were checked again for completeness before data entry and during the data cleaning process.

### 2.8. Data Processing and Analysis

Data were cleaned, coded, and entered into Epi-info version 7 and then exported to SPSS version 25.0 for analysis. Descriptive statistics including frequencies, proportions, mean, and SD was computed and displayed by using tables, charts, and texts. Multicollinearity was checked. Model adequacy was checked by using Hosmer and Lemeshow with 0.21 and 0.456 for knowledge and practice, respectively. Bivariable and multivariable logistic regression analyses were computed to examine the association between the dependent variable and independent variables. Variables with *p* < 0.05 at multivariable logistic regression analysis were considered statistically significant.

## 3. Results

### 3.1. Sociodemographic Characteristics of Respondents

A total of 412 participants were included in the analysis with a response rate of 97.4%. Among respondents, 202 (49.0%) were female and 210 (51.0%) were male. The mean age of the participants was 31.6 years +5.1 standard deviations. Most of the participants 311 (75.5%) of the nurses had a bachelor's degree, nearly 223 (54.1%) were married (Table 1).

### 3.2. Work-Related Characteristics

Most of the participants 180 (43.7%) had 6–10 years of working experience, out of 412 nurses who participated in the study, 120 (29.1%) nurses were working in surgical units and only 153 (37.1%) of nurses responded that they have received formal training on the prevention of DVT. Among all participants, only 141 (34.2%) participants responded to the presence of DVT prevention guidelines in their hospital, and only 221 (53.6%) of them were read professional literature about DVT prevention (Table 2).

### 3.3. Nurses' Knowledge regarding Deep Venous Thrombosis Prevention

From all 412 study participants, more than half 55.6% 95% CI (51.0, 60.4) of the respondents were found to have good knowledge, while 44.4% of the respondents were found to have poor knowledge regarding DVT prevention.

Participants were asked 34 questions to assess their knowledge on the prevention of DVT, and they were categorized into two groups based on their score with their mean. The mean score was 22.8 (SD = ±4.43). Among all questions “DVT occurs as a result of stasis of blood (venous stasis), vessel wall injury, and altered blood coagulation” was the most frequently answered question (97.3%) whereas “Alcohol may predispose to DVT” was the least frequently answered (Table 3).

### 3.4. Nurses' Practice regarding Prevention of DVT

By using 13 practice-based questions, the mean practice score of the respondents was found to be 20.19 (SD = ± 4.84). From all questions, “ encouraging early ambulation surgical patients (70.4%)” was the most frequently answered question on the contrary “Using of the graduated compression stockings” was the least frequently answered question (Table 4).

### 3.5. Factors Associated with Nurses' Knowledge towards DVT Prevention

Nurses working in the medical ward [AOR 3.18, 95% CI (1.42, 7.11)] are more likely to have good knowledge on the prevention of DVT as compared to nurses who worked in the Gyn-obs ward.

Being BSc degrees [ AOR = 3.25, 95% CI (1.245,8.469)] and a master's degree [AOR = 3.48, 95% CI (1.22, 9.89)], working experience ≥11 years [AOR = 2.11; 95%CI (1.07,4.16)], and nurses whoever took training [AOR = 1.59; 95%CI (1.03, 2.47)] were shown to have a strong statistical association during multivariable analysis (Table 5).

### 3.6. Factors Associated with Nurses' Practice towards DVT Prevention

Having a good knowledge about DVT [AOR = 1.74, 95% 3CI (1.15, 2.65)] and working experience ≥11 years [AOR = 3.44, 95% CI (1.46, 8.13)] were significantly associated with practices of nurses towards DVT prevention (Table 6).

## 4. Discussion

The study has attempted to assess knowledge, practice, and associated factors of nurses on DVT prevention, and it revealed that only 55.6% with 95% CI (50.6, 60.4) of the study participants had good knowledge about DVT prevention. This finding is consistent with a study conducted in Kochi, India, and São Paulo, which is 58% and 53.3% of the respondents had good knowledge, respectively [12,13]. Even though there is a difference in socioeconomic status and level of health sector development, the possible reason for this similarity might be using a similar study population (staff nurse), study unit, and study design.

The result of this study is lower than the studies conducted in China with 72.8% and 68.9%, respectively [14,15]. This might be due to differences in the study setting and the use of data collection tools. In this study, nurses working in different wards were included, and a tool with 34 knowledge questions has been employed; whereas in a study that was done in China, the study subjects were only orthopedic nurses and the tool had only 9 knowledge questions.

However, the result of this study was higher than the studies conducted in Zagazig University of Egypt, 27.5% [16] and Port said hospitals of Egypt. 28.9% [17]. This might be due to differences in sample size on which only 90 staff nurses were included in a study conducted at port said hospitals of Egypt, And differences in the tool used (merely about thromboembolism prophylaxis) and differences in cut point as good, average, and poor.

In a multivariable logistic regression analysis, variables like work experience, having training, working unit, and academic qualification were found to have a significant effect on nurses' knowledge regarding DVT prevention.

Working in the Medical ward was found to be 3.175 times more likely to have good knowledge as compared to nurses working at Gyn-obs [AOR 3.175, 95% CI (1.42, 7.11)]. This finding was consistent with the study conducted in South Korea [18]. However, it differ from a study conducted in the University Hospital of China in which the higher scorer was ICU nurses [19]. This might be explained that nurses working in medical wards may encounter DVT cases that can sensitize nurses' knowledge about the prevention of DVT.

Being master and BSc degree in academic qualification was found to be 3.48 and 3.25 times more likely to have good knowledge about DVT prevention as compared to nurses who are diploma [AOR = 3.48, 95% CI (1.22, 9.89)], [AOR = 3.25, 95% CI (1.25, 8.47)], respectively. This finding is in line with the study conducted in Peking Union Medical College Hospital of China [20]. This could be attributed to the possibility of an increase in academic qualification increases the exposure to different academic disciplines, which directly or indirectly help nurses to develop a theoretical background of knowledge on the prevention of DVT.

Study participants whose working experience was greater or equal to 11 years were found to be 2.11 times more likely to be knowledgeable about DVT prevention as compared to nurses whose working experience is less than 5 years [2.11, 95% CI (1.07, 4.16)]. This finding was consistent with the study done in Cyprus, Turkey [9]. The possible explanation might be, the increased years of working experience may offer chances to staff nurses to acquire knowledge from their coworkers and ultimately excel in professional development about patient care [21].

Nurses who took training related to DVT prevention were found to be 1.59 times more likely to have good knowledge than those who had not taken any training [AOR = 1.59, 95% CI (1.03, 2.47)]. This finding was in line with the study conducted in China [12]. The possible explanation might be training may sensitize nurses to retain and ensure a consistent background of knowledge [22].

The result of this study showed that only 48.8% with 95% CI (43.9, 53.7) had good practice about DVT prevention. This finding was consistent with the study conducted in São Paulo that was 44% [13]. On the contrary, the finding of the study is higher than the study conducted in Amrita Institute of medical sciences of India that was 14% [12]. This might be due to the sampling difference on which convince sampling method and only 100 nurses were included a study conducted in Amrita institute of medical science of India. And lower than a study conducted in China (55.4%) [19]. This discrepancy may be due to the study conducted in China has only assessed the practice of nurses exclusively on prophylactic prevention of DVT.

In a multivariable logistic regression analysis, variables like work experience and having good knowledge were found to have significantly associated with the practice of nurses towards DVT prevention.

Nurses with work experience of ≥11 years were found to be 3.44 times more likely to have good practice when compared to those with work experience of <5 years [AOR = 3.44, 95% CI (1.46, 8.13)]. The reason might be nurses with more years of working experience would have more chances to learn from their coworkers. Moreover, a greater year of experience creates a chance for nurses to work in different wards that help nurses to interact and act appropriately as compared to less experienced nurses [23].

Nurses who had good knowledge related to DVT prevention were found to be 1.75 more times to have good practice compared to those who had poor knowledge [AOR = 1.75, 95% CI (1.15, 2.65)]. The finding of this study is in line with the study conducted in port said hospitals of Egypt [17], and the possible justification could be having a theoretical background of knowledge enabled nurses to put their knowledge into practice [24]. Whereas it contradicted with the studies conducted in the intensive care units of Amrita Institute of Medical Sciences, Kochi, India [12]. This might be the difference in sample size and the study population where only ICU nurses with convenience sampling techniques were included in the study conducted in Amrita Institute of Medical Sciences, Kochi, India.

## 5. Conclusion

This study revealed that the knowledge and practice of nurses working in different wards of Amhara region comprehensive specialized hospitals, Northwest, Ethiopia, were not good enough for DVT prevention.

Having higher educational status, attending formal training, being more experienced, and working in medical wards showed a positive and significant association with good knowledge of nurses on DVT prevention; on the other hand, having good knowledge about DVT and higher working experience were found to be associated with good practice of nurses on prevention of DVT.

## 6. Strength and Limitations of the Study

A self-reported questionnaire measure of knowledge and practice of nurses on prevention of DVT is prone to social desirability bias and recall bias. Despite these limitations, this study clearly showed the knowledge, practice, and associated factors of nurses towards DVT prevention among nurses working at the comprehensive specialized hospitals for the first time in Ethiopia.

## Figures and Tables

**Table 1:** Sociodemographic characteristics of nurses working at Amhara region comprehensive specialized hospitals, Northwest, Ethiopia, 2021 (*n* = 412).

Variables	Category	Frequency(*n*)	Percent
Sex	Male	210	51.0
	Female	202	49.0
Age	≤25 years	27	6.6
	26–30 years	181	43.9
	31–35 years	129	31.3
	≥36 years	75	18.2
Marital status	Single	172	41.7
	Married	223	54.1
	Others^∗^	17	4.1
Educational status	Masters	78	18.9
	BSc degree	311	75.5
	Diploma	23	5.6

*∗* Separated, divorced, and widowed.

**Table 2:** Work-related factors about a study participant in Amhara region comprehensive specialized hospitals, Northwest, Ethiopia 2021 (*n* = 412).

Variables	Category	Frequency (*n*)	Percent
Working hospital	UoGCSH	98	23.8
	TGCTH	77	18.7
	FHCSH	120	29.1
	DTCSH	59	14.3
	DMCSH	58	14.1
Working ward	Medical	87	21.1
	Surgical	120	29.1
	Intensive care unit (ICU)	89	21.6
	Emergency	75	18.2
	Gyn-obs	41	10.0
Work experience	≤5 years	177	43.0
	6–10 years	180	43.7
	≥11 years	55	13.3
Training	Yes	153	37.1
	No	259	62.9
Literature reading	Yes	221	53.6
	No	191	46.4
Guideline/protocol	Yes	141	34.2
	No	271	65.8

**Table 3:** Nurses' knowledge on DVT prevention working at Amhara region comprehensive specialized hospitals, Northwest, Ethiopia 2021 (*n* = 412).

Statements on general knowledge, risk factors, and prevention of DVT	True/false	Correctly answered		Incorrectly answered	
		N	%	N	%
1. DVT occurs as a result of stasis of blood (venous stasis), vessel wall injury, and altered blood coagulation.	(T)*∗*	401	97.3	11	2.7
2. Venous thromboembolism (VTE) is a fatal complication of DVT.	(T)*∗*	385	93.4	27	6.6
3. DVT occurs most frequently in the veins of the lower extremities.	(T)*∗*	306	74.3	106	25.7
4. There is no relationship between cancer or cancer treatment and DVTE/VTE.	(F)*∗*	195	47.3	217	51.7
5. There is no relationship between respiratory disease and DVT.	(F)*∗*	232	56.3	180	43.7
6. DVT also occurs frequently in the upper limbs.	(F)*∗*	215	52.2	197	47.8
7. There is no relationship between family history of DVT/VTE and DVT.	(F)*∗*	211	51.2	201	48.8
8. Prolonged immobilization predisposes to DVT in hospitalized patients.	(T)*∗*	347	84.2	65	15.8
9. VTE is a major cause of sudden death in hospitalized patients.	(T)*∗*	308	74.8	104	25.2
10. Surgical patients are more prone than medical patients to DVT/VTE.	(T)*∗*	302	73.3	110	26.7
11. Indwelling intravenous devices such as central venous catheters may predisposes to DVT.	(T)*∗*	279	67.7	113	32.3
12. Paralysis, paresis, or recent plaster cast on lower extremities may predispose to DVT.	(T)*∗*	309	75.0	103	25.0
13. Obesity may predispose to DVT.	(T)*∗*	325	78.9	87	21.1
14. Low body mass index may predispose to DVT.	(F)*∗*	204	49.5	208	50.5
15. Advancing age may predispose to DVT.	(T)*∗*	293	71.1	119	28.9
16. Previous DVT/VTE history may predispose to DVT.	(T)*∗*	301	73.1	111	26.9
17. Major surgery may predispose to DVT.	(T)*∗*	289	70.1	123	29.9
18. Varicose veins may predispose to DVT.	(T)*∗*	297	72.1	115	27.9
19. Exercises may predispose to DVT.	(F)*∗*	219	53.2	193	46.8
20. Trauma may predispose to DVT.	(T)*∗*	306	74.3	106	25.7
21. Smoking may predispose to DVT.	(T)*∗*	268	65.0	114	35.0
22. Alcohol may predispose to DVT.	(F)*∗*	119	28.9	293	71.1
23. Cardiac diseases may predispose to DVT.	(T)*∗*	298	72.3	114	27.7
24. Infections or inflammations may predispose to DVT.	(T)*∗*	295	71.6	117	28.4
25. Pregnancy or postpartum may predispose to DVT.	(T)*∗*	297	72.1	115	27.9
26. Oral contraceptives or hormonal replacement therapy may predispose to DVT	(T)*∗*	257	62.4	115	37.6
27. Foot and leg exercises may prevent DVT	(T)*∗*	333	80.8	79	19.2
28. Elevating legs is necessary to prevent DVT/VTE.	(T)*∗*	322	78.2	90	21.8
29. Early ambulation after surgery may prevent DVT development.	(T)*∗*	308	74.8	104	25.2
30. Bed rest is necessary after major surgery to prevent DVT	(F)*∗*	217	52.7	195	47.3
31. Heparin or low-molecular-weight heparin (LMWH) may prevent DVT development.	(T)*∗*	295	71.6	117	28.4
32. Fluid restriction is necessary to prevent DVT.	(F)*∗*	176	42.7	236	57.3
33. Elastic compression stockings may prevent DVT development.	(T)*∗*	257	62.4	155	37.6
34. The use of intermittent pneumatic compression devices may prevent DVT development	(T)*∗*	241	58.5	171	41.5

*∗* Correct answer.

**Table 4:** Nurses' practice on DVT prevention working in Northwest Amhara region referral hospitals, 2021 (*n* = 412).

Statements on DVT prevention practice	Always		Sometimes		Never	
	N	%	N	%	N	%
1. Providing information to patients and/or relatives about risks and prevention of DVT.	266	64.5	133	32.3	13	3.2
2. Encouraging patients to do foot and leg exercises by themselves or relatives help if patients are unable to do so.	255	61.9	149	36.2	8	1.9
3. Encouraging early ambulation surgical of patients.	290	70.4	105	25.5	17	4.1
4. Assessing the DVT risks of patients regularly.	241	58.5	154	37.4	17	4.1
5. Administering anticoagulants as preventive in clinic	231	56.1	156	37.8	25	6.1
6. Monitoring the side effects of the anticoagulants.	238	57.8	150	36.4	24	5.8
7. Educating the patients on anticoagulants.	253	61.4	139	33.7	20	4.9
8. Educating the patients to avoid injury.	249	60.5	146	35.4	17	4.1
9. Encouraging patients to do elevate legs.	262	63.6	134	32.5	16	3.9
10. Educating the patients on sufficient fluid intake.	253	61.4	143	34.7	16	3.9

**Table 5:** Bivariable and Multivariable analysis of factors associated with nurses' knowledge regarding prevention of DVT working in Amhara region comprehensive specialized hospitals, 2021 (*n* = 412).

Variables	*Knowledge*		COR (95% CI)	AOR (95% CI)	*p* value
	Good	Poor			
	N	N			
Age					
≤25 years	13	14	0.654 (0.270, 1.583)	1.725 (.591, 5.029)	
26–30 years	99	82	0.851 (0.493, 1.467)	1.470 (.711, 3.042)	0.298
31–35 years	73	56	0.918 (0.516, 1.635)	1.457 (0.737, 2.879)	0.278
≥36 years	44	31	1	1	1
Sex					
Male	116	94	0.972 (0.659, 1.434)	1.073 (0.69, 1.699)	0.755
Female	113	89	1	1	1
Marital status					
Single	86	86	1	1	1
Married	133	90	1.475(0.989, 2.207)	1.210 (.739, 1.980)	0.448
Others	10	7	1.429 (0.520, 3.926)	1.282 (0.404, 4.070)	0.674
Working hospital					
UoGCSH	53	45	0.892 (0.464, 1.716)	.644 (0.311, 1.334)	0.236
TGCSH	46	31	1.124 (0.563, 2.243)	1.489 (.696, 3.185)	0.305
FHCSH	63	57	0.837 (0.445,1.574)	0.770 (.377, 1.569)	0.471
DTCSH	34	25	1.030 (0.495, 2.144)	0.980 (.444, 2.166)	0.961
DMCSH	33	25	1	1	1
Working ward					
Medical	64	23	3.92 (1.995, 8.595)	3.175 (1.417,7.113)	0.005*∗*
Surgical	73	47	2.19 (1.066, 4.511)	1.872 (0.88,3.943)	0.099
ICU	39	50	1.10 (0.521, 2.329)	0.966 (0.448,2.083)	0.930
Emergency	36	39	1.30 (0.604, 2.811)	1.092 (0.496,2.406)	0.827
Gyn-obs	17	24	1	1	1
Educational status					
Masters	45	33	3.117 (1.152, 8.433)	3.48 (1.223, 9.898)	0.019*∗*
BSc degree	177	134	3.019 (1.208, 7.547)	3.248 (1.245, 8.469)	0.016*∗*
Diploma	7	16	1	1	1
Work experience					
≥11 years	39	16	2.356(1.227, 4.524)	2.109(1.069,4.162)	0.031*∗*
6–10 years	100	80	1.208 (0.797, 1.832)	1.248 (0.806, 1.931)	0.321
1–5 years	90	87	1	1	1
Attend training on prevention of DVT					
Yes	101	52	1.988 (1.314, 3.007)	1.598 (1.034, 2.47)	0.035*∗*
No	128	131	1	1	1
Availability of guideline/protocol					
Yes	84	57	1.281 (0.848, 1.934)	1.110 (.810, 2.117)	0.271
No	145	126	1	1	1
Reading professional literature related to DVT					
Yes	125	96	1.091 (0.622, 1.356)	0.879 (0.555, 1.392)	0.583
No	104	87	1	1	1

*∗* Significant at *P* < 0.05.

**Table 6:** Bivariable and multivariable analysis of factors associated with nurses' practice regarding prevention of DVT working in Amhara region comprehensive specialized hospitals, 2021 (*n* = 412).

Variables	Practice		COR	AOR	*p* value
	Good	Poor			
	N	N			
Age					
≤25 years	6	21	0.301 (0.270,1.583)	0.468 (0.145,1.489)	0.204
26–30 years	91	90	1.038 (0.493,1.467)	1.689 (.817,3.493)	0.178
31–35 years	67	62	1.101 (0.516,1.635)	1.494 (.768,3.011)	0.248
≥36 years	37	38	1	1	1
Sex					
Male	98	112	0.841 (0.571, 1.238)	0.825 (529, 1.286)	0.396
Female	103	99	1	1	1
Marital status					
Single	77	95	1	1	1
Married	118	105	1.387 (0.930, 2.067)	0.954 (.574, 1.555)	0.852
Others	6	11	0.673 (0.238, 1.902)	0.553 (0.160, 1.915)	0.350
Working hospital					
UoGCSH	50	48	1.196 (0.624, 2.292)	1.163 (.575, 2.353)	0.675
TGCSTH	44	33	1.531 (0.771, 3.038)	1.441 (0.691, 3.007)	0.33
FHCSH	40	80	0.574 (0.303, 1.089)	0.555 (0.272, 1.134)	0.106
DTCSH	40	19	2.417 (1.140, 5.124)	1.705 (0.743, 3.913)	0.208
DMCSH	27	31	1		1
Working ward					
Medical	45	42	2.066 (0.956, 4.465)	1.657 (.702, 3.912)	0.258
Surgical	66	54	2.357 (1.126, 4.935)	2.114 (.932, 4.797)	0.073
ICU	38	51	1.437 (0.665, 3.104)	1.601 (.688, 3.685)	0.278
Emergency	38	37	1.981 (0.900, 4.357)	1.854 (.787, 4.3681)	0.158
Gyn-obs	14	27	1	1	1
Educational status					
Masters	27	34	0.610 (0.286, 1.864)	0.419 (.147, 1.196)	0.097
BSc degree	161	167	0.717 (0.306, 1.683)	0.444 (.171, 1.151)	0.104
Diploma	13	10	1	1	1
Work experience					
≥11 years	40	15	3.799 (1.955, 7.384)	3.443 (1.457, 8.133)	0.005*∗*
6–10 years	88	92	1.363 (0.897, 2.070)	1.215 (.732, 2.018)	0.460
1–5 Years	73	104	1	1	1
Attend training on prevention of DVT					
Yes	144	151	1.199 (0.804, 1.789)	1.416 (.876, 2.289)	0.155
No	57	60	1	1	1
Availability of guideline/protocol					
Yes	66	75	0.887 (0.590, 1.333)	1.033 (.630, 1.694)	0.897
No	135	136	1	1	1
Reading professional literature related to DVT					
Yes	120	101	1.613(1.092,2.384)	1.498 (.955,2.349)	0.079
No	81	110	1	1	1
Knowledge on DVT prevention					
Good	128	101	1.910 (1.287, 2.833)	1.745 (1.149, 2.651)	0.009*∗*
Poor	73	110	1	1	1

*∗* Significant at *P* < 0.05.

## Data Availability

All data are available upon reasonable request and the readers could contact the corresponding author.

## Abbreviations

AOR: Adjusted odds ratio
COR: Crude odds ratio
CDC: Centers for Disease Control
CMHS: College of Medicine and Health Science
DMCSH: Deberemarkos Comprehensive Specialized Hospital
DTCSH: Debretabor Comprehensive Specialized Hospital
DVT: Deep venous thrombosis
FHCSH: Felegehiot Comprehensive Specialized Hospital
HA-VTE: Hospital-acquired venous thromboembolism
ICU: Intensive care unit
PE: Pulmonary embolism
TGSTH: Tibebegion Specialized Teaching Hospital
UoGCSRH: University of Gondar Comprehensive Specialized Referral Hospital
VTE: Venous thromboembolism.
